# Modified endoscopic transnasal orbital apex decompression in dysthyroid optic neuropathy

**DOI:** 10.1186/s40662-021-00238-2

**Published:** 2021-04-28

**Authors:** Yunhai Tu, Mingna Xu, Andy D. Kim, Michael T. M. Wang, Zhaoqi Pan, Wencan Wu

**Affiliations:** 1 The Eye Hospital of Wenzhou Medical University, No. 270 Xueyuan Xi Road, Wenzhou, Zhejiang, 325027 P. R. China; 2 Department of Ophthalmology, New Zealand National Eye Centre, The University of Auckland, Auckland, New Zealand

**Keywords:** Endoscopic transnasal orbital decompression, Dysthyroid optic neuropathy, Thyroid-associated ophthalmopathy, Visual acuity, Visual field

## Abstract

**Background:**

To describe the surgical technique and assess the clinical efficacy and safety of modified endoscopic transnasal orbital apex decompression in the treatment of dysthyroid optic neuropathy.

**Methods:**

In this retrospective research, forty-two subjects (74 orbits) who underwent modified endoscopic transnasal orbital apex decompression for the treatment of dysthyroid optic neuropathy were enrolled. Preoperative and postoperative best-corrected visual acuity (BCVA), visual field mean deviation (MD), Hertel exophthalmometry, and new onset diplopia were assessed before and after the intervention. The Wilcoxon test was used for differential analysis. Linear mixed-models’ analyses were conducted to assess the potential predictors for BCVA change.

**Results:**

Postoperatively, the mean BCVA improved from 0.70 ± 0.62 logMAR to 0.22 ± 0.33 logMAR. BCVA significantly improved in 69 eyes (93%), remained stable in 4 eyes (5%) and deteriorated in 1 eye (1%). MD of visual fields improved from −13.73 ± 9.22 dB to −7.23 ± 7.04 dB. Proptosis decreased from 19.57 ± 3.38 mm to 16.35 ± 3.01 mm. Preoperative BCVA, MD of visual fields and medical rectus diameter were independent factors associated with improvements in BCVA (*P* < 0.05) by linear mixed-models’ analyses. Eighteen patients (42.9%) developed new diplopia postoperatively.

**Conclusion:**

Modified endoscopic transnasal orbital apex decompression effectively restores vision in dysthyroid optic neuropathy.

**Supplementary Information:**

The online version contains supplementary material available at 10.1186/s40662-021-00238-2.

## Background

Dysthyroid optic neuropathy (DON) is a vision-threatening complication of thyroid-associated ophthalmopathy (TAO) [1]. Accumulation of hyaluronic acid in extraocular muscles and orbital fatty connective tissues leads to volume expansion and subsequent compression of the orbital apex, causing optic nerve ischemia and inhibition of the axonal nerve flow [1].

Several treatment options are currently available for DON. Intravenous glucocorticoid (IV-GC) can reduce orbital tissue swelling, and has been shown to effectively restore visual function in up to 42.5% of the patients with DON [2]. However, high-dose steroids can lead to severe complications such as acute liver injury and hypertension [2]. Besides, relapse of orbitopathy is a common consequence after steroid withdrawal, and optic nerve damage can ensue [3]. Radiotherapy may reduce orbital inflammation but it currently lacks robust evidence of efficacy [4], and thus is reserved for poor surgical candidates unresponsive to steroid therapy [1]. Surgical removal of the orbital wall aims to expand the available orbital volume and decompress the optic nerve [5]. This can be performed in conjunction with orbital fat decompression if fat hypertrophy is present [6]. The medial orbital wall can be removed via transcutaneous, transconjunctival, transcaruncular, or transnasal approaches [5]. The transcutaneous approach is relatively less complex to perform but leaves patients with visible scarring. Although scarring is less prominent with the transconjunctival approach, this procedure is limited by the restricted exposure of the medial wall [7]. A malleable retractor can secure a wider surgical view, but this is at the functional risk of increased retrobulbar pressure [8]. The endoscopic transnasal approach provides good visualization [9, 10], and has been shown to provide superior postoperative visual recovery than the transcaruncular approach [11]. For these reasons, endoscopic transnasal medial wall decompression has gained progressive popularity [6, 12, 13].

To resolve optic nerve compression, an extensive posterior decompression must be achieved. Inadequate decompression at the orbital apex leading to disease recurrence is commonly reported [12, 14]. Repeated surgical procedures are technically difficult given the presence of scars and obliteration of the anatomical landmarks [14]. This study aimed to combine the endoscopic transnasal approach with modified orbital apex decompression to achieve a more extensive posterior decompression and assess its clinical efficacy and safety in the treatment of DON.

## Materials and methods

### Participants

This was a retrospective review of patients with DON who underwent endoscopic transnasal orbital apex decompression at Wenzhou Medical University Eye Hospital from Jan. 2017 to Dec. 2019. The institution’s ethics committee waived informed consent for the extraction of data from hospital records without revealing personal identifiers. Patients with TAO were deemed eligible if their age was greater than or equal to 18 years and with the presence of at least one of the followings: visual acuity (VA) > 0.1 logMAR; visual field (VF) defect (defined as the mean deviation (MD) of less than −5 dB); optic disc swelling; evidence of orbital apex crowding on a high-resolution computed tomography (HRCT) scan. A minimum follow-up of 1 month was required for inclusion and patients with a history of previous ocular surgery, glaucoma, and visual defects from other ocular conditions were excluded from the study.

### Clinical measures

All participants underwent a comprehensive ocular examination including the determination of best-corrected visual acuity (BCVA), degree of proptosis, intraocular pressure (IOP), and optical coherence tomography retinal nerve fibre layer (RNFL) thickness. Ocular motility was assessed at the nine cardinal positions of gaze. Fundus photographs and a HRCT image of the orbit were obtained. The VF test was performed with a Humphrey automated perimeter and the 30–2 program using the Swedish Interactive Threshold Algorithm (SITA) standard. TAO was graded using the Clinical Activity Score [15]. Blood samples for thyroid function tests were collected on the day before the surgery. HRCT scan was performed in all patients within 3 months of surgery. The diameter of the medial rectus muscle was determined by axial HRCT scans. Follow-up data were collected at 2 weeks, 1, 3, 6, 12, and 24 months postoperatively.

### Surgical technique

**Fig. 1:**
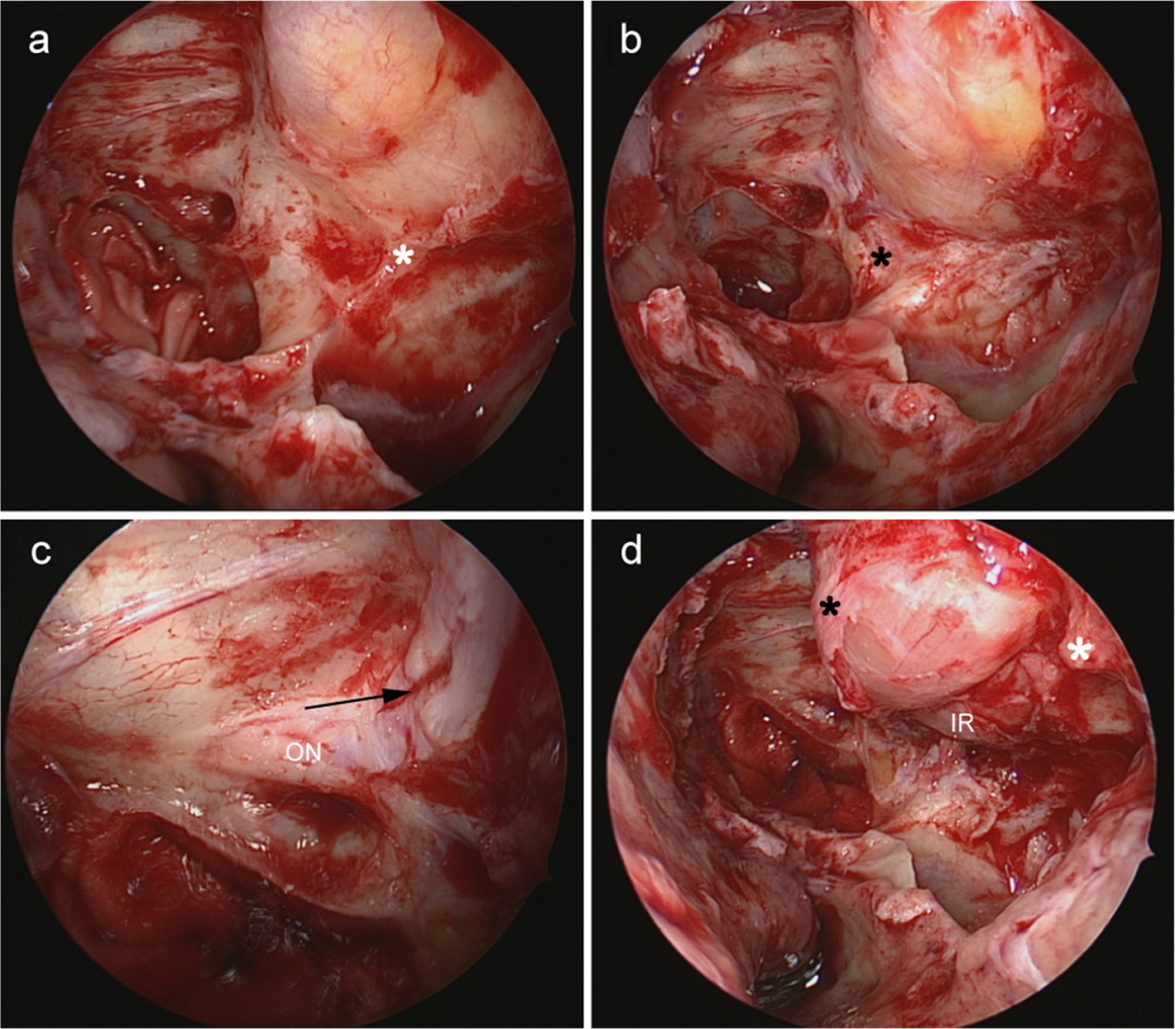
Endoscopic transnasal orbital apex decompression was performed in a DON patient. **a** Before removal of the inferior wall of the left orbital apex (white asterisk). **b** After removal of the bone at the junction of orbital apex and pterygopalatine fossa (black asterisk). **c** After decompression of the anterior segment of the optic canal. The orbital periosteum of the orbital apex is incised (black arrow). **d** Preservation of the inferomedial strut (white asterisk) and creation of periosteal band (black asterisk). IR = inferior rectus, ON = optic nerve

**Fig. 2:**
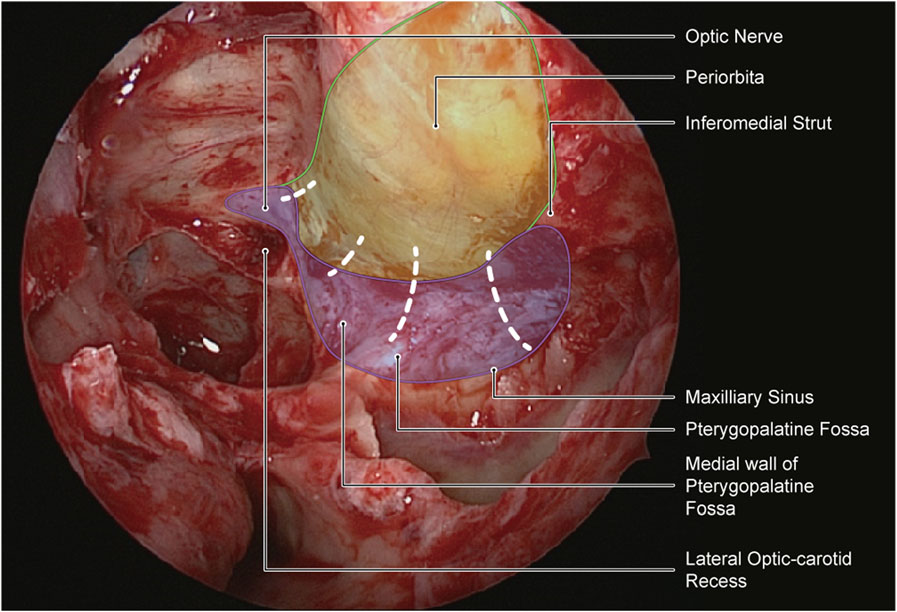
Left nostril endoscopic endonasal views. The purple area indicates the extent of modification. The green area indicates the extent of ordinary bone resection.The periosteum was incised along the white dotted line

All of the surgical procedures were performed by two experienced ophthalmologists (WW and YT). The surgical procedure was performed under general anaesthesia, with the patient in a supine position and the head elevated to 30 degrees. The nasal cavity was sterilized with 0.05% povidone-iodine and packed with a gauze soaked in 1% lidocaine (1:1000 epinephrine) to achieve vasoconstriction. The endoscopic total sphenoethmoidectomy was performed [6, 16], where the sphenoid sinus was accessed with a 45-degree endoscope (Karl Storz, Tuttlingen, Germany) 1 to 1.5 cm above the choana. The posterior wall of the ethmoid sinus was removed with a tricut blade (4.0 mm; 1,884,004; Medtronic Inc., USA) and a high-speed round diamond bur (5 mm; 1885061HS; Medtronic Inc., USA) to allow visualization of the optic canal and the internal carotid artery. The maxillary sinus was accessed via the upper edge of the inferior turbinate or the maxillary ostium. The maxillary sinus antrostomy was performed for better visualization of the posterior wall. An incision at the lateral wall of the nasal cavity was made to remove the lamina papyracea, the anterior part of the optic canal and the medial wall of the pterygopalatine fossa. The protrusion of the optic canal and the medial and anterior walls of the pterygopalatine fossa (Fig. 1a and b) were ground thin using a microdrill (XPS3000; Medtronic Inc., USA) at a rotational speed of 6000 rpm, and removed carefully with a freer septum elevator. Care was taken to avoid damage to the optic nerve, the ethmoidal arteries, and the sphenopalatine artery. Then, the periorbita of the orbital apex and the annulus of Zinn were incised with a sharp 20G MVR knife (MVR-Lance 20G, MANI, Japan) (Fig. 2). The posterior wall of the maxillary sinus was removed beneath the inferomedial strut by 3-4 mm along with the lamina papyracea. The inferomedial strut was preserved to prevent inferomedial displacement of the eyeball (Fig. 1d). The superior and inferior periorbita were incised from the orbital apex to the maxillary line using the 20G MVR knife, creating a band parallel to the medial rectus muscle (Fig. 1d). Extraconal and intraconal fat were removed with a low suction cutting instrument (the New Direction Medical Optic Instrument Co, Ltd., Dezhou, PR China). The fat was removed gently and under direct vision to avoid disruption of blood vessels. The amount of fat removed during the surgery was guided by the requirement to achieve an appropriate reduction in proptosis. Given the danger and difficulty in intraconal fat decompression, we do not suggest this to be routinely performed in patients with smaller amounts of proptosis. Orbital soft tissue herniation was covered with a bioresorbable nasal dressing (Merogel, Medtronic Xomed, USA) soaked in triamcinolone acetonide.

The conventional endonasal orbital decompression was defined as described by Chu et al. [17], the medial orbital wall was decompressed from the face of the sphenoid posteriorly to the basal lamella anteriorly. The superior margin consisted of the skull base with the orbital floor as the inferior margin. They did not perform a sphenoidotomy. However, in the modified surgery that we performed here, decompression was undertaken from the posterior lacrimal crest posteriorly to the minor wing of sphenoid and anterior part of the optic canal. The inferior margin was the line between the posterior wall of the maxillary sinus and the medial wall of the pterygopalatine fossa. The periosteum was also incised to further reduce the pressure in the orbital apex. Moreover, all patients were combined with extraconal and intraconal fat decompression.

Postoperatively, all the patients received 3 days of intravenous methylprednisolone (500 mg) and 5 days of broad-spectrum antibiotics. Patients were advised to refrain from any strenuous physical activity and nose blowing for 14 days after the surgery. On the 14th postoperative day, the remaining dressings in the nasal cavity were removed.

### Outcome measures

Postoperative improvement, stabilization and deterioration of DON were defined in accordance with the criteria used in previous studies [13, 18]. Significant improvement of DON was defined as either an improvement of BCVA by at least 0.2 or a final BCVA > 0.5 logMAR with improved or normal VFs. Stabilization was defined as the final BCVA ± 0.1 logMAR in comparison to the baseline while deterioration was a worsening of BCVA by at least 0.2 logMAR. The following standards were used for the estimation of VA: counting fingers equals the decimal acuity of 0.014 and detecting hand motion equals the decimal acuity of 0.005.

### Statistical analysis

Data analysis was performed with IBM SPSS 26.0 software (SPSS Inc., Chicago, IL, USA) and R 3.5.3 (Butler, Cullis, Gilmour, & Gogel, 2009). Wilcoxon test was used for statistical analysis of continuous pre- and post-operative parameters. The postoperative parameters were taken at the last follow-up. Summary statistics are expressed as mean and standard deviation when data were normally distributed, whereas median and interquartile range were used for data with non-normal distribution. For the evaluation of the effect of predictors of interest on BCVA change, linear mixed-effect models (adjusting for within-patient inter-eye correlations, where appropriate) were fitted using the ‘lmerTest’ package of R. Patient ID was included as a random term to account for inter-eye correlation. Regression coefficients were used for evaluating the association effect. Univariate models were used to test potential predictors individually. Multivariate models were adjusted for age, body mass index, sex, and smoking history. Decimal scores were converted into logMAR units for statistical analysis. *P*-value of < 0.05 was considered statistically significant.

## Results

### Patient characteristics

A total of 74 eyes from 42 patients were included in this study (Table. 1). The median follow-up time was 6 months (range: 2–8 months). Patient demographics and baseline thyroid function are outlined in Table 1. The median duration of DON was 6 months (range: 2–11 months). Thirty-two patients had bilateral and 10 patients had unilateral DON. All patients were euthyroid before the surgery.

**Table 1:** Patient characteristics

Characteristics	*N* = 42
Female (%)	18 (43)
Age (years)	57.30 ± 9.29
BMI (kg/m^2^)	24.40 ± 2.71
Follow up time (months)	6 (2–8)
Medical background	
Diabetes (%)	6 (14)
Hypertension (%)	15(36)
Thyroid	
Hyperthyroidism (%)	38 (90)
Hypothyroidism (%)	1 (2)
Normal (%)	3 (7)
Duration of Thyroid dysfunction (months)	12 (7.75–36)
Duration of TAO (months)	10.5 (6–12)
Duration of DON (months)	6 (2–11)
Serum	
FT3 (pg/ml)	5.27 (4.44–5.94)
FT4 (ng/dl)	17.25 (13.5–20.94)
TSH (μIU/ml)	0.94 (0.08–3.78)
TG (ng/ml)	43.47 (10.44–181)
TGAb (IU/ml)	12.05 (10–18.44)
TPOAb (IU/ml)	21.55 (11.32–56.16)

*BMI*= body mass index, *TAO*= thyroid associated ophthalmopathy, *DON*= dysthyroid optic neuropathy, *FT3*= free triiodothyronine, *FT4*= free thyroxinem, *TSH*= thyroid stimulating hormone, *TG*= thyroglobulin, *TGAb*= thyroglobulin antibody, *TPOAb*= thyroid peroxidase antibody

### Outcomes

**Fig. 3:**
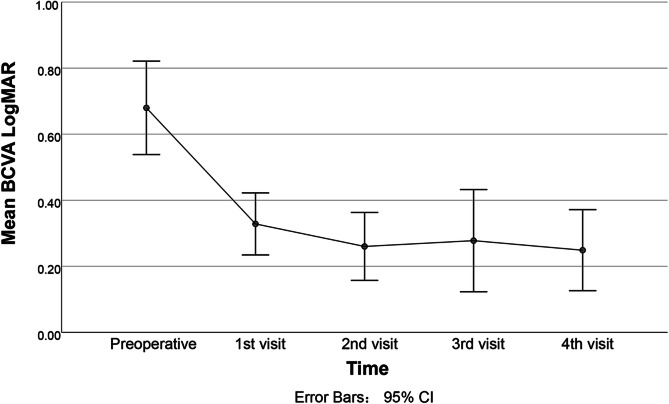
Mean change in best-corrected visual acuity (BCVA) from baseline to postoperative visits 1–4. Five waves of data were collected, including baseline, the follow-up visits occurred in 2 weeks, 1, 3, 6 months, respectively

The mean BCVA improved from 0.70 ± 0.62 logMAR to 0.22 ± 0.33 logMAR postoperatively (*P* < 0.001), with a mean change of −0.50 ± 0.62 logMAR (Table 2). According to the defined outcome measures, 69 of 74 eyes (93.2%) had significant improvements in the BCVA. BCVA remained stable in 4 eyes and deteriorated in 1 eye due to postoperative central retinal artery occlusion (CRAO). Furthermore, 8 of the 26 eyes with a 1.0 logMAR > preoperative BCVA ≥ 0.3 logMAR, and 10 of the 22 eyes had a preoperative BCVA ≥ 1.0 logMAR achieved a BCVA < 0.1 logMAR postoperatively. The changes in mean BCVA during the follow-up are shown in Fig. 3.

**Table 2:** Overview of preoperative and postoperative visual outcomes

Eyes (*N* = 74)	Preop	Postop	*P* value^a^
Best corrected visual acuity (logMAR)			
Mean ± SD	**0.70 ± 0.62**	**0.22 ± 0.33**	< 0.001^***^
Visual field			
VFI (%)	**65.86 ± 32.48**	**83.98 ± 21.14**	< 0.001^***^
MD (dB)	**−13.73 ± 9.22**	**−7.23 ± 7.04**	< 0.001^***^
PSD (dB)	**6.26 ± 3.44**	**5.15 ± 2.92**	< 0.001^***^
Missing data (%)	15 (20)	22 (30)	
Proptosis measurement (mm)			
Mean ± SD	**19.57 ± 3.38**	**16.35 ± 3.01**	< 0.001^***^
Missing data (%)	–	13 (19)	
RNFL of ONH (*N* = 39) (μm)			
Temporal	**82.31 ± 42.41**	**71.89 ± 12.15**	0.001***
Superior	**141.96 ± 49.41**	**129.89 ± 25.80**	0.006^**^
Nasal	88.76 ± 34.55	86.74 ± 32.47	0.312
Inferior	**139.35 ± 52.83**	**125.87 ± 29.99**	0.047^*^
Missing data (%)	–	36 (48)	
Medial rectus muscle diameter (mm)			
Mean ± SD	8.53 ± 2.42	9.51 ± 2.22	0.096
Missing data (%)	8 (11)	34 (46)	
Fundus			
Papilledema (%)	17 (22)	5 (7)	
Papillary pallor (%)	6 (8)	1 (1)	
Large Cup/Disk ratio (%)	7 (9)	1 (1)	
Normal (%)	59 (79)	67 (91)	

Values with statistical significance are shown in bold

*VFI*= visual field index (Humphrey), *MD*= mean deviation, *PSD*= pattern standard deviation, *RNFL*= retinal nerve fibre layer, *ONH*= optic nerve head

*P < 0.05, **P < 0.01, ***P < 0.001

^a^Paired Wilcoxon test

Baseline MD values of the VFs were available for 59 eyes. Sixteen eyes had mild (MD ≥ −6 dB), 16 eyes had moderate (−6 dB > MD ≥ −12 dB) and 27 eyes had severe (MD < −12 dB) VF defects. Postoperative MD values of VFs were available for 52 eyes, of which 41 eyes have available VFs examination before surgery. The MD of VF improved from −13.73 ± 9.22 dB to −7.23 ± 7.04 dB postoperatively (*P* < 0.001), with a mean MD improvement of 7.07 ± 9.01 dB. VF improved in 33 eyes (80.5%), of which 10 eyes (24.4%) achieved an MD ≥ −2 dB. No significant improvements were seen in 8 eyes (19.5%). Preoperative BCVA and MD of VFs were independent factors associated with improvements in BCVA on the multivariate regression analyses (Table 3) (B = 0.08, *P* < 0.001; B = −0.04, *P* < 0.001, respectively). No significant correlation was found between the preoperative thyroid stimulating hormone (TSH) level and the improvement in BCVA (*P* = 0.083).

**Table 3:** Predictive factors on the change of BCVA

	Univariate model		Multivariate model	
	Estimate (95% CI)	*P* value	Estimate (95% CI)	*P* value
Baseline age, per year older	−0.01 (−0.03, 0.00)	0.139	–	–
Gender: Male	0.17 (−0.14, 0.46)	0.294	–	–
Smoking history: never	0.17 (−0.51, 0.84)	0.632	–	–
TSH level, per 1 uIU/ml higher	−0.02 (−0.04, 0.00)	0.083	–	–
BMI, per 1 higher	−0.01 (−0.07, 0.05)	0.719		
IVGC therapy: never	−0.11 (−0.45, 0.23)	0.524	–	–
Duration of DON, per month longer	0.00 (0.00, 0.00)	0.944	–	–
Preop. CAS, per 1 score higher	−0.05 (−0.18, 0.06)	0.928	–	–
Medium rectus diameter, per 1 mm thicker	**0.08 (0.02, 0.13)**	0.008**	**0.07 (0.01, 0.13)**	0.019*
Preop. BCVA, per 0.1 LogMAR higher	**0.08 (0.07, 0.09)**	< 0.001***	**0.08 (0.07, 0.09)**	< 0.001***
Preop. MD, per 1 dB greater	−**0.03 (**−**0.04, −0.01)**	< 0.001***	**−0.04 (−0.05, −0.02)**	< 0.001***
Preop. exophthalmos, per 1 mm higher	0.04(0.00, 0.09)	0.051	–	–

Confounding factors including age, gender, body mass Index, duration of DON and smoking history were adjusted for

Values with statistical significance are shown in bold

*BCVA*= best-corrected visual acuity, *TSH*= thyroid stimulating hormone, *BMI*= body mass index, *IVGC*= intravenous glucocorticoid, *DON*= dysthyroid optic neuropathy, *CAS*= clinical activity score, *MD*= mean deviation

*P < 0.05; **P < 0.01; ***P < 0.001

At the follow-up visits, proptosis decreased from 19.57 ± 3.38 mm to 16.35 ± 3.01 mm (*P* < 0.001), with a mean reduction of 3.50 ± 2.53 mm. The RNFL thickness decreased in the temporal, superior, and inferior quadrants from 82.31 ± 42.41 μm to 71.89 ± 12.15 μm (*P* = 0.001), 141.96 ± 49.41 μm to 129.89 ± 25.80 μm (*P* = 0.006), and 139.35 ± 52.83 μm to 125.87 ± 29.99 μm (*P* = 0.047), respectively. No significant change was noted in the nasal quadrant (88.76 ± 34.55 μm to 86.74 ± 32.47 μm, *P* = 0.312).

The mean diameter of the medial rectus muscle was greater postoperatively, however, this difference was not statistically significant (8.53 ± 2.42 mm vs. 9.51 ± 2.22 mm, *P* = 0.096). The preoperative medial rectus diameter was also an independent predictor of BCVA improvement in the multivariate modelling (Table 3) (B = 0.073, *P* = 0.019).

### Complications

Three eyes (4.05%) had recurrent optic neuropathy due to the reactivation of TAO. BCVA was effectively restored with a 1-week course of IV-GC treatment. Thirteen patients (30.95%) had preoperative diplopia, of which 8 had complete resolution postoperatively. Eighteen patients (42.9%) developed new diplopia postoperatively, of which 9 patients (21.4%) recovered following strabismus surgery. The remaining 9 patients were managed conservatively. One eye (1.35%) developed CRAO requiring treatment with IOP lowering drugs and nitroglycerine. At 6 months postoperatively, this eye had a BCVA of counting fingers at 20 cm. Sphenoid sinusitis occurred in 2 patients (4.76%).

## Discussion

We have described a technique that combined the endoscopic transnasal approach with modified orbital apex decompression in patients with DON. Our finding suggest that the modified orbital apex decompression may be an effective technique for VA improvement.

Bony decompression remains the mainstay treatment for steroid-refractory DON [19] and is perceived as safe and effective, however, treatment failures are commonly reported (Supplementary additional file 1) [14, 20–22]. Indeed, in previous studies, 7–28% of patients undergoing orbital decompression required repeat procedures for persistent orbitopathy [20–22]. Repeat surgery is difficult due to the presence of scars and subsequent obliteration of anatomical landmarks [14], and carries a significant risk of intraorbital injuries [23]. Inadequate decompression at the orbital apex is the commonest cause of persistent or recurrent optic neuropathy [14, 21].

Previous studies [9, 12] have confirmed the benefits of optic canal decompression, however, the depth of the decompression and inclusion of the optic nerve sheath remains controversial [24, 25]. The incision of the optic nerve sheath poses a risk of cerebral spinal fluid leakage and nerve damage [24]. The extent of the dissection on the lateral wall of the sphenoid might depend on the optic canal. In this study, a combined decompression of only the anterior part of the optic canal (Fig. 1c) was performed to further alleviate the apical pressure while minimizing the risk of injury to the optic nerve fibres. Anterior optic canal decompression provides ample room for further decompression of Annulus of Zinn, and to reduce optic disc swelling [26]. Additionally, the anterior and medial walls of the pterygopalatine fossa were removed for a similar effect. With the resections of the posterior wall of the maxillary sinus, the volume of the orbital apex can be expanded allowing inward and downward movement of the orbital contents, and thus attenuating the stress in the orbital apex.

The findings of this study support the combined use of the above modifications (Fig. 3). After the surgical procedure, 93.2% of eyes had significant improvements in BCVA with a mean improvement of 0.48 logMAR. This is comparable to that reported in the earlier studies where an average improvement ranging from 0.3 logMAR to 0.55 logMAR was observed following transnasal endoscopic orbital decompression [6, 27, 28]. Our finding also advocates the use of the described surgical intervention even in those with poor baseline BCVA. Lastly, an improvement in the VF parameters was observed, similar to that previously reported (Table 2) [28].

Our results suggest that poorer preoperative VA, severer VF defect and larger medial rectus diameter are likely positive predictive factors for the improvements in BCVA. Wang et al. [29] suggested that the patients with better preoperative VA had more significant VA improvement. A possible reason for this discrepancy could be a ceiling effect where eyes with good VA have lesser VA gains. The diameter of the medial rectus muscle is already known to be an independent predictor of optic neuropathy [30]. To the best of our knowledge, this study is the first to suggest the preoperative diameter of the medial rectus muscle as a predictor of surgical success.

Notably, recurrent optic neuropathy was only observed in 3 eyes in this study, and all were successfully treated with IV-GC. We aimed to minimize the recurrence of optic neuropathy and improve the post-VA by adequate decompression of the orbital apex. The follow-up time was relatively short and studies with longer follow-up periods are recommended to validate the conclusion of the present study. An alternative explanation may be that there was an insignificant increase in the mean postoperative medial rectus thickness (8.53 ± 2.42 mm vs. 9.51 ± 2.22 mm, *P* = 0.096). We observed that the thickness of the medial rectus increased after surgery, although this difference was not statistically significant. A similar observation had already been reported [20]. In the patients with the thickening of the medial rectus, none showed a severe visual loss, which can indirectly prove the effectiveness of the modification procedure.

The mean reduction in proptosis was significant (3.49 mm) albeit lesser than the reported studies (2.6 mm to 6.2 mm) [6, 27, 31]. This, however, may be due to the smaller baseline proptosis (19.57 mm) in our study population, as the amount of reduction achievable is proportional to the degree of preoperative proptosis [32]. Furthermore, a greater reduction in proptosis is associated with an increased risk of postoperative diplopia [32, 33] and does not necessarily result in superior visual outcomes [14, 34], and thus it was not the primary goal of this study. Remarkably, the reduction in proptosis benefited from the extraconal and intraconal fat decompression here, which was useful to retract the eyeball and mitigate the orbital apex crowding. Fat decompression is often used in patients with greater amounts of preoperative proptosis, particularly those with fat expansion [35]. However, Prat et al. suggested that even those with muscle expansion or smaller amounts of proptosis appreciated benefit from fat decompression [36].

RNFL thickness also decreased in all quadrants, except the nasal, and this finding is similar to that reported by Park et al. [37] and although the exact reasons remain unclear, it may be assumed to have developed from the regression of the optic disc oedema.

The most common complication is the development of new or worsening of pre-existing diplopia [12, 19, 34]. The reported incidence of diplopia following transnasal endoscopic orbital decompression varies between 11.7 and 81.2% [6, 12, 34]. In this study, the inferomedial strut and horizontal strip of periorbita were preserved to minimize such risks [38]. However, 18 patients (42.9%) developed new diplopia postoperatively. In DON, preserving the VA should be prioritized, as secondary diplopia can often be managed with prism corrected lenses or strabismus surgery [12, 39]. Out of 18 patients, 9 underwent strabismus surgery with good effect, and the eyes of the remaining 9 patients opted for conservative management. One patient developed a CRAO, presumably due to ophthalmic arterial spasm following intraoperative haemorrhage and the release of serotonin [40, 41]. Despite the prompt initiation of treatment, this patient only achieved partial visual recovery.

## Conclusions

In conclusion, endoscopic medial orbital decompression combined with modified orbital apex decompression effectively restores vision in DON. Adequate decompression at the orbital apex is crucial to minimize the recurrence of compressive optic neuropathy.

## Supplementary Information

**Additional file 1.** A 76-year-old woman presented to our institution with signs of recurrent dysthyroid optic neuropathy. Preoperative BCVA was OD: counting fingers at 20 cm and OS: counting fingers at 40 cm. After undergoing bilateral medial orbital decompression at a local hospital, her BCVA improved to counting fingers at 80 cm bilaterally. One month postoperatively, the BCVA decreased to counting fingers at 10 cm bilaterally, refractory to corticosteroid treatment. Orbital CT scan demonstrated an enlarged medial rectus muscle causing compression of the optic nerve (a). In previous surgery, the medial and inferior walls of the orbital apex were not removed (b). The patient subsequently underwent endoscopic transnasal orbital apex decompression (c, d). In this procedure, the medial wall (a, bold arrow) and the inferior wall (b, outlined arrow) of the orbital apex were removed. BCVA improved to OD: 20/40 and OS: 20/20 postoperatively

## Data Availability

The datasets used and/or analyzed during the current study are available from the corresponding author on reasonable request.

## Abbreviations

BCVA: Best-corrected visual acuity
CRAO: Central retinal artery occlusion
DON: Dysthyroid optic neuropathy
FT3: Free triiodothyronine
FT4: Free thyroxine
HRCT: High-Resolution Computed Tomography
IVGC: Intravenous glucocorticoid
MD: Mean deviation
ONH: Optic Nerve Head
PSD: Pattern standard deviation
RNFL: Retinal nerve fibre layer
SITA: Swedish interactive threshold algorithm
TAO: Thyroid associated ophthalmopathy
TG: Thyroglobulin
TSH: Thyroid stimulating hormone
VFI: Visual field index (Humphrey)
