# Supplementary figures and images for: Modified endoscopic transnasal orbital apex decompression in dysthyroid optic neuropathy
Source: Eye Vis (Lond). 2021 Apr 28;8:19. doi: 10.1186/s40662-021-00238-2 (PMC8080388; doi:10.1186/s40662-021-00238-2)

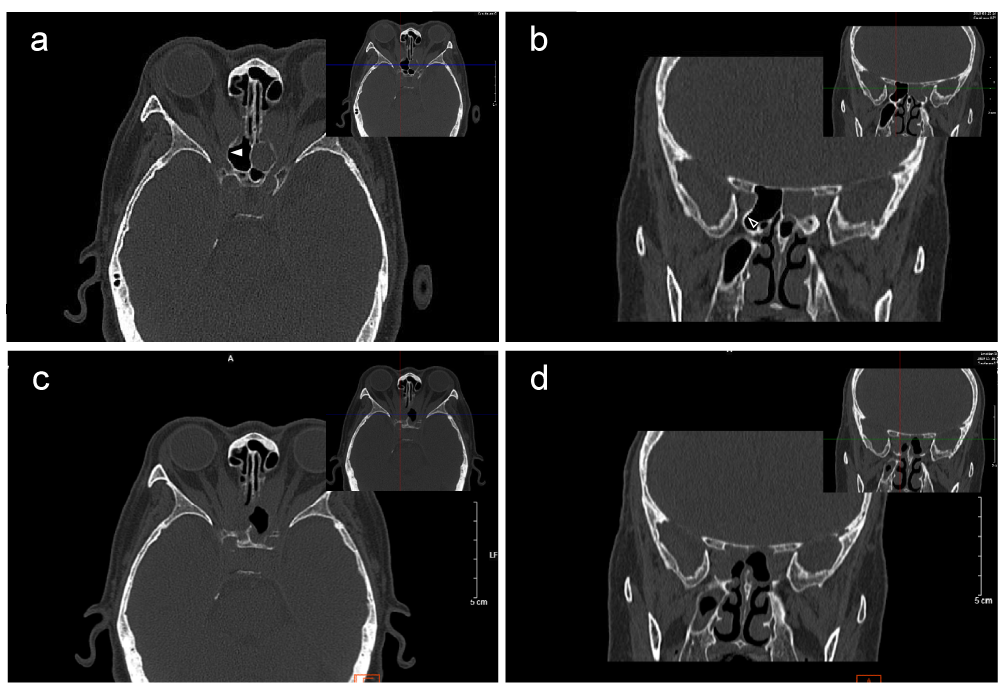

Supplement: Supplementary file 1 — Additional file 1. A 76-year-old woman presented to our institution with signs of recurrent dysthyroid optic neuropathy. Preoperative BCVA was OD: counting fingers at 20 cm and OS: counting fingers at 40 cm. After undergoing bilateral medial orbital decompression at a local hospital, her BCVA improved to counting fingers at 80 cm bilaterally. One month postoperatively, the BCVA decreased to counting fingers at 10 cm bilaterally, refractory to corticosteroid treatment. Orbital CT scan demonstrated an enlarged medial rectus muscle causing compression of the optic nerve (a). In previous surgery, the medial and inferior walls of the orbital apex were not removed (b). The patient subsequently underwent endoscopic transnasal orbital apex decompression (c, d). In this procedure, the medial wall (a, bold arrow) and the inferior wall (b, outlined arrow) of the orbital apex were removed. BCVA improved to OD: 20/40 and OS: 20/20 postoperatively [file 40662_2021_238_MOESM1_ESM.tif]
